# Oral Tolerance Induced by OVA Intake Ameliorates TNBS-Induced Colitis in Mice

**DOI:** 10.1371/journal.pone.0170205

**Published:** 2017-01-18

**Authors:** Lisiery N. Paiatto, Fernanda G. D. Silva, Julia Bier, Márcia R. Brochetto-Braga, Áureo T. Yamada, Wirla M. S. C. Tamashiro, Patricia U. Simioni

**Affiliations:** 1 Department of Genetics, Evolution and Bioagents, Institute of Biology, State University of Campinas, UNICAMP, Campinas, São Paulo, Brazil; 2 Institute of Biosciences, Universidade Estadual Paulista, UNESP, Rio Claro, São Paulo, Brazil; 3 Faculty of Food Engineering, University of Campinas, UNICAMP, Campinas, São Paulo, Brazil; 4 Department of Histology and Embryology, Institute of Biology, State University of Campinas, UNICAMP, Campinas, São Paulo, Brazil; 5 Department of Biomedical Science, Faculty of Americana, FAM, Americana, São Paulo, Brazil; Wayne State University, UNITED STATES

## Abstract

**Introduction:**

Literature data have shown that the consumption of dietary proteins may cause modulatory effects on the host immune system, process denominated oral tolerance by bystander suppression. It has been shown that the bystander suppression induced by dietary proteins can improve inflammatory diseases such as experimental arthritis. Here, we evaluated the effects of oral tolerance induced by ingestion of ovalbumin (OVA) on TNBS-induced colitis in mice, an experimental model for human Crohn’s disease.

**Methods and Results:**

Colitis was induced in BALB/c mice by instilling a single dose of TNBS (100 mg/kg) in ethanol into the colon. Tolerized mice received OVA (4mg/mL) dissolved in the drinking water for seven consecutive days, prior to or concomitantly with the intrarectal instillation. Control groups received protein-free water and ethanol by intrarectal route. We observed that either the prior or concomitant induction of oral tolerance were able to reduce the severity of colitis as noted by recovery of body weight gain, improvement of clinical signs and reduction of histological abnormalities. The *in vitro* proliferation of spleen cells from tolerant colitic mice was lower than that of control mice, the same as the frequencies of CD4^+^ T cells secreting IL-17 and IFN-γ. The frequencies of regulatory T cells and T cells secreting IL-10 have increased significantly in mice orally treated with OVA. The levels of inflammatory cytokines (IL-17A, TNF-α, IL-6 and IFN-γ) were lower in supernatants of cells from tolerant colitic mice, whereas IL-10 levels were higher.

**Conclusion:**

Our data show that the modulation of immune response induced by oral tolerance reduces the severity of experimental colitis. Such modulation may be partially attributed to the increase of Treg cells and reduction of pro-inflammatory cytokines in peripheral lymphoid organs of tolerant mice by bystander suppression.

## Introduction

Recently, inflammatory bowel disease (IBD) has been receiving more attention from physicians and researchers due to the increase in its incidence in human populations [1–5]. IBD comprises a set of related diseases, collectively called Crohn's Disease (CD) and Ulcerative Colitis (UC), which origin has been attributed to the breakdown of tolerance to self-antigens in the intestinal mucosa [6]. The mechanisms involved in these autoimmunities are diverse and cannot be considered mutually exclusive. They may include a combination of factors among which one can highlight the unbalanced production of cytokines and interleukins such as interleukin (IL)-9 IL-10, IL-35, transforming growth factor (TGF)-β. Several other molecules, transcription factors and receptors are associated with colitis, including Cytotoxic T-Lymphocyte Antigen (CTLA)-4, Leukocyte Activation Gene (LAG) -3, indoleamine (IDO), perforin/antagonists and Glucocorticoid-Induced Tumor Necrosis Factor Receptor (GITR) [7–11].

Recent literature, however, emphasizes the role of Th17 cells in inflammatory diseases such as colitis. T helper (Th) 17 cells and other IL-17-producing-cells play a crucial role in intestinal inflammatory diseases. IL-17 together with IL-22 appear to be related to induction of colitis, since these cytokines initiate and amplify the local inflammatory signs and promote the activation of regulatory mechanisms directly against the cells of the intestinal epithelium [12,13]. In turn, IFN-γ induces the production of inflammatory cytokines by cells of the innate immune system, contributing to an increased tissue inflammation seen in colitis [4,12,14,15]. Therefore, modulation of Th17 and INF-γ-secreting cells may affect the inflammation in colitis.

On the other hand, IL-10 produced by macrophages and regulatory T cells may skew the response into a regulatory one, leading to a reduction in inflammation [15]. In addition, several results suggest that tolerogenic dendritic cells (DC) lead to the generation of induced regulatory T (Treg) cells (CD4^+^CD25^+^Foxp3^+^) and an increase in the number of lymphocytes expressing the suppressor molecule CTLA-4 [16–19].

Accordingly, several studies have attempted to modulate the inflammatory response observed in experimental colitis by the induction of a tolerogenic response [16,20–23]. Specifically, oral tolerance by dietary antigens administration is a tool to the modulation of the immune system. The suppression of immune response on oral tolerance is due to the low responsiveness of local or systemic immune system, triggered by such protein antigens [24–28]. Despite the great interest in the subject, the effects of oral tolerance by bystander suppression in colitis are not yet fully understood [16,21,29,30].

The aim of this study was to evaluate the effects of oral tolerance on TNBS-induced colitis in mice of BALB/c. Tolerance was induced by oral administration of ovalbumin (OVA) in the previous and subsequent induction of colitis. Parameters as body weight, histology of target tissues, cell proliferation, cytokine production and expansion of regulatory CD25+Foxp3+ T cells and Th17 cells on spleen cell cultures were evaluated here. Our data suggested that oral tolerance to OVA modulates the immune responses against TNBS in BALB/c mice by bystander suppression. Suppression of the immune response is reflected in the increase in regulatory T cells and decreased production of pro-inflammatory cytokines that together markedly ameliorated the severity of the colitis.

## Materials and Methods

### Animals

SPF female BALB/c mice (four weeks old) were obtained from the Multidisciplinary Center for Biological Research (CEMIB) of the University of Campinas (UNICAMP) Campinas, SP, Brazil, and housed in plastic cages in groups of five. They were maintained in specific pathogen-free environment at 25°C ±1 and photoperiod of 12/12 hours, and fed with autoclaved Nuvilab CR-diet and water *ad libitum* for at least 4 weeks before being used in experiments. The methods described in this manuscript were carried out in accordance with the ‘Guide for the Care and Use of Laboratory Animals’, as promoted by the Brazilian College of Animal Experimentation (COBEA), and was approved by the Ethics Committee for Animal Experimentation of University of Campinas. (CEUA/UNICAMP. Protocol #3077–1). All experimental procedures were performed under proper anesthesia and all efforts were made to minimize animal suffering. Mice general health was daily monitored for signs of inflammation such as rectal swelling, rectal bleeding, soft stool or weight loss. On day 5 after TNBS instillation, all mice were sacrificed by cervical dislocation after anesthesia with a mixture of ketamine (60 mg/kg, Ketalar; Pfizer) and xylazine (6 mg/kg, Rompun; Bayer) (i.p.).

### Oral tolerance

**Fig 1:**
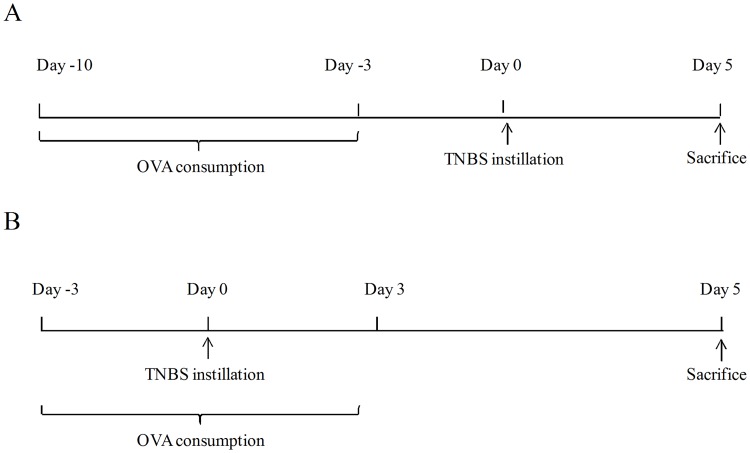
Flowchart of the TNBS-induced colitis and oral tolerance design. Panel A: Previous oral tolerance. Panel B: Concomitant oral tolerance.

The induction of oral tolerance to OVA was performed as described elsewhere [31] and depicted in Fig 1. Briefly, 4mg/mL OVA (Rhoster Commerce and Industry Ltda, Vargem Grande Paulista, SP, Brazil) was added to water supply of BALB/c mice for 7 consecutive days. The control mice received protein-free water.

### Induction of experimental colitis

Experimental colitis was induced in BALB/c mice according to indications of Neurath and colleagues [29,32]. Mice were anesthetized and instilled with 100 μL of 1 mg/mL TNBS (2,4,6—trinitrobenzenesulfonic acid; Sigma, USA) solution in 50% ethanol by intrarectal route. Control animals received 100 μL of 50% ethanol (Fig 1).

### Evaluation of clinical signs of colitis

All groups were weighed daily until sacrifice. Clinical signs such as diarrhea, rectal prolapse, bleeding and cachexia were registered and assigned as scores, ranging from 0 to 2, with 0: no change, 1: slight change, and 2: severe change.

### Histological analysis

On 5 days after TNBS instillation, mice were euthanized and the distal portion of the large intestines were removed and fixed with 4% paraformaldehyde. The pieces were cleared and embedded in paraffin. The sections were stained with hematoxylin and eosin. For the histological analysis, the sections were evaluated for the presence of folds, hemorrhage, infiltration of leukocytes on two distal portions of the intestines (1 to 2 cm and 2 to 3 cm from the rectum). The thickening of the wall of the colon was measured in micrometers in distal portions, with Infinity Analyze Nikon H600L program (100X). A score ranging from 0 to 20 was assigned [32,33].

### Spleen cell proliferation

On day 5 after colitis induction, mice of all groups were sacrificed and spleens were aseptically removed. The spleens were macerated individually and erythrocytes in cell suspensions were lysed. Cells were pelleted at 200 g for 10 min and cell concentration was adjusted to 1 x 10^6^ cells/mL in RPMI medium (Sigma, USA) supplemented with 10% fetal bovine serum (Cultilab). Cells were stained with 1.25μM Carboxyfluorescein diacetate succinimidyl ester (CFSE) according to manufacturer's instructions (Invitrogen, USA). To determine the maximum uptake, aliquots of the cell suspensions stained with CFSE were fixed with 1% formaldehyde and analyzed by flow cytometer. Stained cells were seeded at 4x10^5^ cell/well in sextuplicate, and Concanavalin A (ConA) was added to each well at final concentration of 2.5μg/mL. Plates were incubated at 37°C in humidified incubator, with 5% CO_2_ for 72 hours. After the incubation period, cells were fixed with 1% formaldehyde and proliferation was assessed in CD4^+^CSFE^+^ cells by flow cytometer [34]. Acquisitions were performed with FACScalibur flow cytometer (Becton-Dickinson) and analyzes were done with the FCS Express 5 Plus Research Edition software (FCS Express Launcher). Results were expressed as proliferation index (fold change) calculated in relation to control group. Cells not stained with CFSE were also cultured in the presence of ConA and their supernatants were collected for dosage of cytokines.

### Phenotypic profile of T lymphocytes

The frequencies of T CD4^+^CD25^+^ Foxp3^+^ (Treg cells), T CD4^+^IL17^+^, T CD4^+^IFN**γ**^+^ and T CD4^+^IL-10^+^ cells in the cultures were assessed by flow cytometer. Briefly, cell suspensions were washed and initially stained with anti-CD4-PE (Clone GK1.5) and anti-CD25-FITC (Clone 7D4). Following, cells were permeabilized by the addition of fixation/permeabilization buffer (Cytofix / Cytoperm fixation/permeabilization kit, Becton-Dickinson, BD) and stained with anti-Foxp3-APC (clone FJK-16S), anti-IL-17-APC (clone eBIO17B7) or Alexa Fluor 647 (Clone TC11-18410), anti-IFN**γ**-APC (Clone XMG1.2), IL-10-APC (clone JESS-16E3) according to manufacturer’s instructions. Acquisitions were performed with FACScalibur flow cytometer and analyzes were done with the FCS Express 5 Plus, Research Edition software.

### Th1/Th2/Th17 cytokines determination

IL-2, IL-4, IL-6, IL-10, IL-17A, IFN-γ and TNF-α were quantified in culture supernatants of spleen cells by flow cytometry by using Multiplex CBA kit (BD Cytometric Bead Array Th1/Th2/Th17, San Diego, USA) according to manufacturer’s instructions. Cells were acquired in FACSCalibur cytometer and analyzed with FCAP Array TM Software Version 3.0 (BD).

### Statistical analysis

The statistical analysis was performed using GraphPad Prism 5 (GraphPad Software, San Diego, CA, USA). The statistical significance of differences between control and experimental groups was determined by one-way ANOVA, followed by multiple comparisons *Bonferroni’s* test. Results were expressed as mean ± Standard Error Mean (SEM). Values were considered significant at P > 0.05. All data are representative of at least three independent experiments.

## Results and Discussion

The gastrointestinal tract is in constant contact with dietary proteins, commensal microorganisms and potentially pathogenic microorganisms. To ensure the maintenance of homeostasis of the organism, the immune system of the intestinal mucosa must be able to tolerate antigens from diet and commensal microbiota and generate protective responses against harmful antigens. The ability of intestinal mucosa-associated lymphoid tissue (MALT) to suppress systemic immune response against ingested proteins is known as oral tolerance. Oral tolerance has been demonstrated in various experimental models and for different antigens [20,24,26,35,36]. Nowadays, other routes are under evaluation in order to develop oral tolerance strategies for immunotherapy. In this sense, eye-induced tolerance is a promising therapeutic tool in the treatment of complicated autoimmune diseases such as CIA [37,38] or EAE [39–41]. Several tolerance protocols are being proposed for controlling inflammatory manifestations, in particular autoimmune diseases such as colitis [42–48].

The TNBS-induced colitis is a widely used experimental model for the study of inflammatory bowel disease (IBD), since their clinical symptoms and immunological response are similar to those observed in human intestinal diseases [33,49–55]. BALB/c mice are often used for induction of colitis by TNBS administration because they develop a milder disease than SJL strain, but with significant clinical and immunological signals [49,54,56].

**Fig 2:**
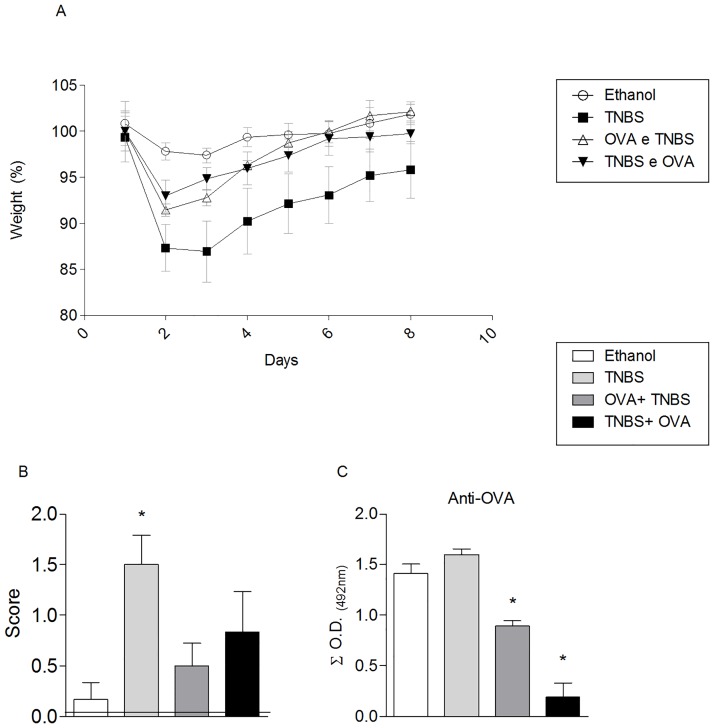
OVA intake improves the severity of TNBS-induced colitis in mice. For induction of tolerance an OVA solution (4mg / ml) was provided in drinking water to BALB/c mice (n = 5) for seven consecutive days, either 3 or 10 days before colitis induction. The experimental colitis was induced in BALB/c mice by intrarectal instillation of TNBS in 50% ethanol (1 mg / mL, 100 μL). Two control groups were included: mice that received protein-free water and were instilled with either ethanol (white bar) or TNBS (light gray bar). Panel A: Temporal changes in body weight, in percentage. All mice were weight daily and the weight alteration was represented by percentage in comparison with the mean initial value found on day 1 and represented as 100%. Body weights in orally treated mice were significantly higher than in non-tolerant mice. Panel B: Clinical signs were evaluated for the presence of diarrhea, rectal prolapse, bleeding and cachexia, assigning a score ranging from 0 to 2, with 0: no change, 1: slight change, and 2: severe change. Panel C: Oral tolerance to OVA modulates antibody production. Oral tolerance and experimental colitis was induced as illustrated in Fig 1. Five days after TNBS instillation, mice of all groups were immunized with OVA (10 mg) in aluminum hydroxide (1 mg) by intraperitoneal route, and 14 days later challenged i.p. with 10mg OVA in saline solution. On day 21, sera were collected, diluted 1: 100 to 1: 12,800 and tested by ELISA for anti-OVA antibodies (A) and anti-TNP (B). Values are means ± SEM sum of the optical densities (O.D.) Data are representative of three independent experiments (n = 5). ANOVA followed by Bonferroni post-test were used to determine statistical significance (p <0.05).

Data presented here show that the consumption of OVA, either prior or concomitant to colitis induction, led to the reduction of the weight (Fig 2A) and of typical clinical signs of TNBS-induced colitis, such as diarrhea, rectal prolapse, bleeding and cachexia (Fig 2B). The subsequent intraperitoneal challenge with OVA led to decreased levels of serum antibodies in animals fed protein, indicating a systemic effect of oral treatment (Fig 2C).

**Fig 3:**
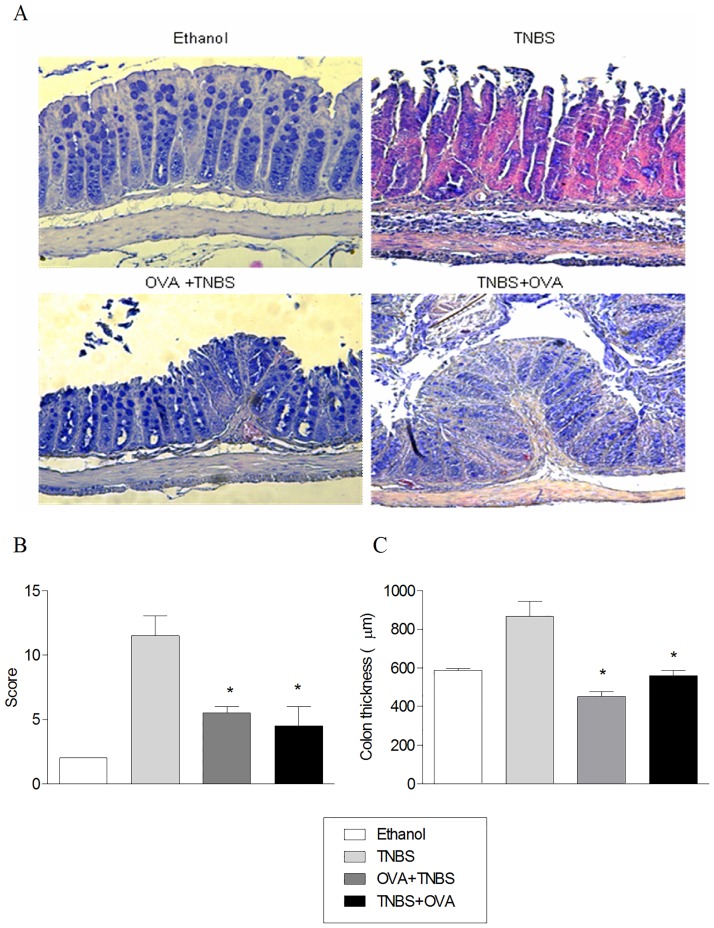
The oral tolerance to OVA changes histopathological features in the large intestine of mice with TNBS-induced colitis. BALB / c mice were exposed to the treatments described in Fig 2 and sacrificed 5 days after induction of colitis. Distal part of large intestine was collected and fixed in paraffin for histological processing. Panel A: Histological sections of 5μm were stained with hematoxylin and eosin and examined by light microscopy (200X). Panel B: Sections were assessed for the presence of folds, hemorrhage, and infiltration of leukocytes at two distal portions of the intestines (1 to 2 cm and 2 to 3 cm from the rectum), assigning a score ranging from 0 to 20. Panel C: Thickening of the colon wall, measured in micrometers in two distal portions was analyzed with Infinity Analyze Nikon H600L program (100X). Two independent analyzes were performed.

To evaluate the possible effects of OVA consumption on the intestinal mucosa, the distal segments of the large intestine of mice from all groups were collected on the fifth day after the induction of colitis and evaluated histologically. As summarized in Fig 3, the oral treatment with OVA prior to or concomitant with induction of colitis was able to partially preserve the integrity of the colonic tissue of mice with colitis. This can be observed by the significant reduction of histological changes (bends, hemorrhage and leukocyte infiltration) in the tissues of animals tolerized to OVA (Fig 3A and 3B) as well as the preservation of the wall thickness of the colon (Fig 3C) compared to the control animals. Together, these results indicate a possible bystander suppression of the immune response in mice tolerized with OVA, able to alter the course of experimental disease.

Previous studies conducted in our laboratory showed that it is possible to induce oral tolerance to OVA in animals that exhibit a normal repertoire of receptors to the target antigen [31,57]. Several mechanisms seem to be involved in the induction of tolerance, including anergy, clonal deletion and induction of Treg cells [24,58–61]. Weiner and colleagues [22,48,62] suggested that the mechanisms that operate in the induction of oral tolerance are directly related to the antigen administration regimen. Thus, administration of high doses of antigens at once would lead to clonal deletion or anergy, while multiple doses of antigen in low concentrations would be capable of promoting the suppression mediated by suppressor/regulators T cells. However, Siewert et al [63] found a high rate of Foxp3^+^ T cells from oral tolerant mice that had been induced by high dose of antigen. This finding indicates that higher doses of antigen did not lead to clonal deletion, but the generation of suppressive responses associated with oral tolerance. Some cytokines, such as granulocyte macrophage colony stimulating factor (GM-CSF) can modulate DC towards a tolerogenic profile acting as an anti-inflammatory or regulatory modulator in autoimmune diseases, depending on its dose and the presence of other cytokines. Tolerogenic DCs can increase the frequency and function of regulatory T-cells [64–69].

Data from our laboratory have shown that continued ingestion of low doses of OVA can interfere with the phenotypic distribution of intraepithelial lymphocytes (IELs) in the small intestine of wild type BALB/c. After ingestion of OVA, BALB/c mice showed increased frequency of CD4^+^Foxp3^+^ regulatory T cells and increased expression of regulatory cytokines on IELs [70].

**Fig 4:**
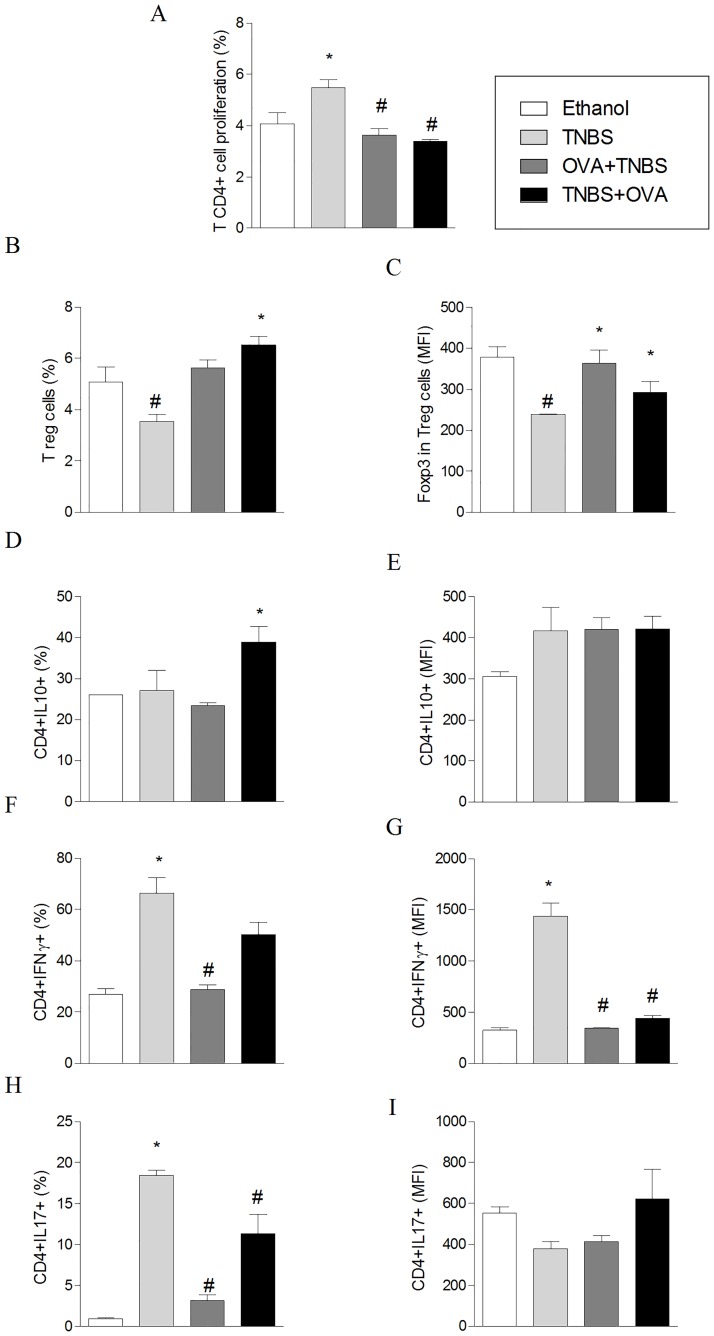
The oral tolerance to OVA alters the proliferative response and cytokine producing cells from mice with TNBS-induced colitis. The induction of tolerance and colitis was performed as described in Fig 2. Mice were sacrificed five days after TNBS instillation. Spleens were aseptically removed; cells were labeled with CFSE and cultured at a concentration of 2x10^6^ cells / ml in the presence of Concanavalin A (ConA; 2,5μg / ml) for 72 hours at 37°C and 5% CO2. Panel A: Spleen cell proliferation. Cells were fixed in 1% formaldehyde and the readings performed in flow cytometer (FACSCalibur, BD). Proliferation was calculated using the software FCS Express and represents the inverse of the ratio of the fluorescence exhibited by the cells after 72 hours of culture and those immediately after labeling with CFSE. The cell frequency (Panel B) and the mean fluorescence intensity (MFI) of T regulatory CD25^+^Foxp3^+^ cells (Panel C), and the frequency of cytokine producing cells (Panels D-I) were monitored within the CD4^+^ T cell gate. The values correspond to the mean ± S.E.M. of two independent experiments (n = 5). ANOVA followed by Bonferroni a posteriori test was used to determine statistical significance.

To investigate the systemic effects of OVA intake on cellular immune response of colitic mice induced by TNBS, spleen cells were collected on the fifth day after the induction of colitis and assessed for their proliferative capacity and for the expansion of CD4^+^ T lymphocytes with regulatory and effector profiles. The results summarized in Fig 4 and in S1 Fig show that the proliferation of spleen cells from mice fed with OVA was significantly lower than that observed in cultures of spleen cells from the control group (Fig 4A), whereas the frequency of Treg cells (TCD4^+^CD25^+^ Foxp3^+^) and expression of Foxp3 was higher in cultures of cells from tolerized mice (Fig 4B and 4C). It was also observed an increase in the frequency of CD4^+^IL-10^+^ T cells in spleen cell cultures of mouse fed with OVA (Fig 4D) as well as a reduced frequency of CD4^+^IFN-γ^+^ T cells and CD4^+^IL17^+^ T cells (Fig 4F and 4H) compared with non-tolerant group.

**Fig 5:**
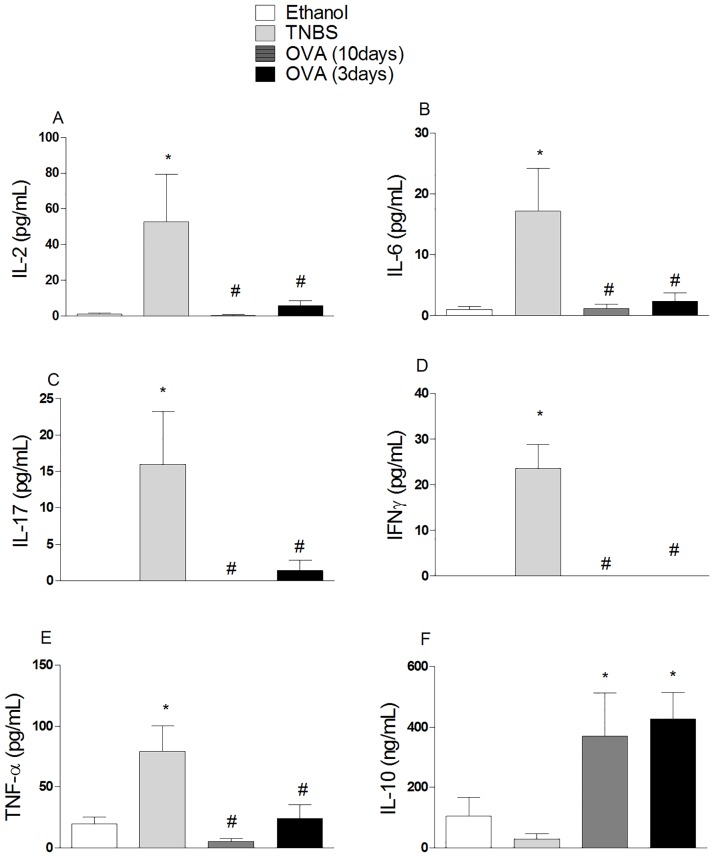
The oral tolerance to OVA alters cytokine levels in supernatants of ConA-stimulated spleen cells from mice with TNBS-induced colitis. Cultures of spleen cells were carried out as described in Fig 4. Cytokine levels were evaluated in the culture supernatants by using the CBA Multiplex kit (Cytometric Bead Array Th1 / Th2 / Th17, BD) and readings were performed in a flow cytometer (FACSCalibur, BD). Cytokine concentrations were determined using the array FCAP TM Version 3.0 Software (BD). Results were expressed as means ± S.E.M. obtained from two independent experiments (n = 5).ANOVA followed by Bonferroni a posteriori test was used to determine statistical significance.

Assessment of cytokines in the supernatants of spleen cells revealed no statistically significant differences in IL-2 secretion in the experimental groups (Fig 5A), but showed an increase in IL-10 levels (Fig 5B) and reduction in levels of IFN-gamma (Fig 5C) and IL-17 (Fig 5D) in the spleen cell cultures from mice tolerized with OVA compared to the non-tolerant group. The results presented here are consistent with data recently obtained by our group in the experimental model of arthritis [34].

Corroborating our data, the literature shows that regulatory T cells may play an important role in autoimmune diseases [15,50,71–75] and the expansion of this population can aid in the control of clinical manifestations [16,76–78]. Moreover, Th17 cells have a potential role in the induction of inflammatory bowel disease and its reduction must also contribute to the improvement of the clinical picture in several autoimmune conditions [12,76,79,80].

It is already known that CD4^+^CD25^+^Foxp3^+^ cells on the intestinal mucosa seems to be able to induce local generation of Th3 cells secreting TGF-β (LAP^+^), type 1 regulatory T (Tr1) cells and CD8^+^T reg cells [48,81]. These regulatory T cells generated in the intestine migrate to secondary lymphoid organs such as Peyer's patches and mesenteric lymph nodes, which inhibit the generation of nonspecific effector cells, by a mechanism known as bystander suppression [22,82]. In addition, the imbalance between proinflammatory and anti-inflammatory cytokines released by the intestinal mucosa determines the intensity and duration of the inflammatory response seen in experimental colitis [14,15,33,83–86].

Data from our laboratory has shown that it is possible to protect mice against experimental arthritis (CIA) by oral tolerization with OVA as a preventive or therapeutic intervention, although the antigens involved in tolerization and triggering the disease had been administered independently [34].

An increased frequency of both CD4^+^FoxP3^+^ and CD4^+^ IL10^+^ lymphocytes was also observed after *in vitro* restimulation of spleen cells from tolerized arthritic animals. These results, however, as well as those of Vaz and colleagues [87–90] show indirect effects of oral tolerance that do not fit perfectly in bystander suppression model.

By definition, the bystander suppression occurs when the immune response to a particular epitope suppresses the response to another epitope administered concurrently or immediately after the first one. The effects of bystander suppression is related to the secretion of cytokines such as TGF-β, IL-4 and IL-10, and by the action of regulatory T cells [42,91,92].

Interventions leading to bystander suppression may be of great interest in autoimmunity regulation. Thus, the suppressive response initiated by mucosal administration of dietary proteins associated with self-antigens has been gaining ground as a preventive or therapeutic intervention proposed for autoimmune diseases [24,42,48]. In this respect, several authors have shown that the oral tolerance to dietary antigens may prevent or inhibit the progression of systemic or organ-specific autoimmune diseases [34,35,93]. In this regard, data from our and others laboratories have shown that it is possible to reduce the immune response to a different antigen from that used to induce oral tolerance, even if this antigen is administered several days after ingestion of tolerogen and parenteral challenge with tolerated antigen [34,38]. Recent work from Vaz group have shown that there is an expansion of regulatory T CD4^+^Foxp3^+^ and T CD4^+^LAP^+^ non-specific cells in the early stage of parenteral exposure to the antigen used in the oral treatment, phenomenon that is not seen in non-tolerant animals subjected to the same treatment [94]. Similarly to what we have shown with our work, other authors have demonstrated that increased IL-10 production correlates with the ability to reduce inflammatory responses associated with human and experimental colitis [95,96].

## Conclusions

Our results indicate that the development of oral tolerance to OVA was able to reduce the signs and the immune response in TNBS-induced colitis. The immunomodulation observed can be attributed to the expansion of regulatory T cells and IL-10 producing cells, by a mechanism known as bystander suppression. Further studies are in progress to evaluate the role of dendritic cells in the protection afforded by oral tolerance in this colitis model.

## Supporting Information

S1 Fig Oral tolerance reduces the proliferation of T lymphocytes, increases the proportion of CD25^+^Foxp3^+^Tregs and reduces IL-17-and INF-γ- producing-T cells on colitic mice. Five days after the TNBS instillation, leukocytes were harvested from spleens of mice that received OVA by oral route, as described in Fig 2. Leukocytes from spleens of naïve and untreated TNBS mice were used as controls. The spleen cells were stained with CFSE and cultured at a concentration of 2x10^6^ cells / mL in the presence of Concanavalin A (ConA; 2,5μg/mL) for 72 hours at 37°C and 5% CO2. CFSE: Cells were fixed in 1% formaldehyde and the readings performed in flow cytometer (FACSCalibur, BD). Cell proliferation was determined using flow cytometry and assessed by fluorescence decay of the probe in the gate of CD4^+^ cells. The frequency of CD25+ Foxp3+ Tregs in the different groups was evaluated in the gate of CD4^+^ cells. IFN-γ-, IL-10-, and IL-17-producing cells were evaluated in the gate of CD4^+^ cells as well. Histograms and plots are derived from a representative animal from two independent experiments (n = 5/each assay).
(TIF)
