# Supplementary figures and images for: Oral Tolerance Induced by OVA Intake Ameliorates TNBS-Induced Colitis in Mice
Source: PLoS One. 2017 Jan 18;12(1):e0170205. doi: 10.1371/journal.pone.0170205 (PMC5242488; doi:10.1371/journal.pone.0170205)

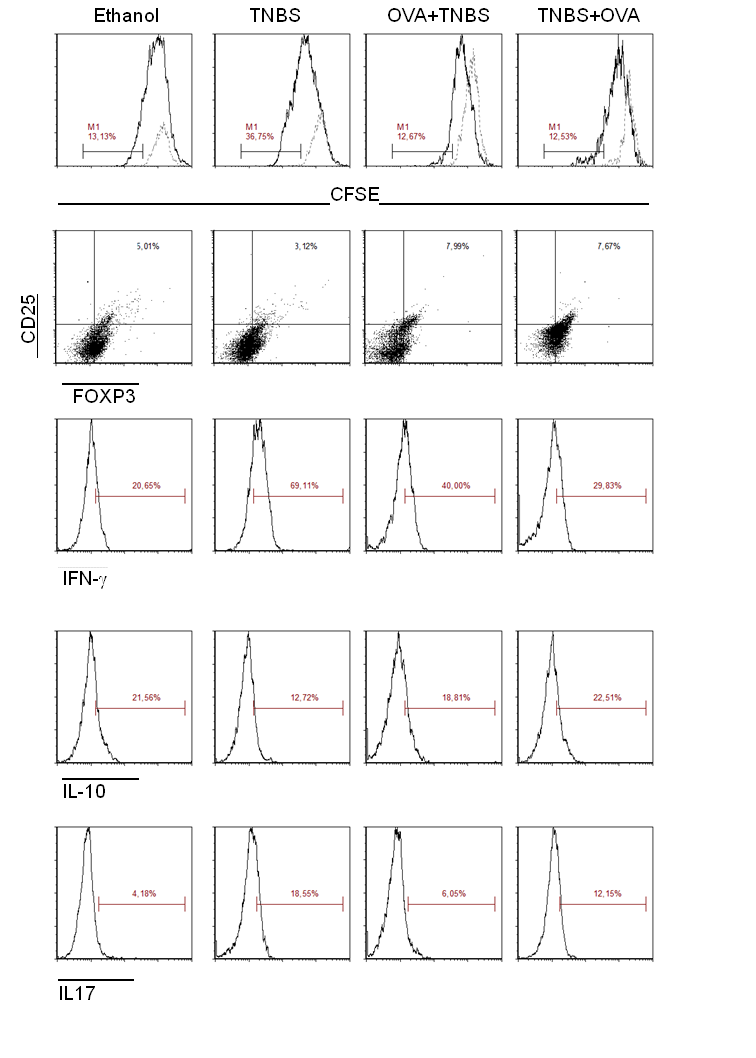

Supplement: S1 Fig — Five days after the TNBS instillation, leukocytes were harvested from spleens of mice that received OVA by oral route, as described in Fig 2. Leukocytes from spleens of naïve and untreated TNBS mice were used as controls. The spleen cells were stained with CFSE and cultured at a concentration of 2x10^6^ cells / mL in the presence of Concanavalin A (ConA; 2,5μg/mL) for 72 hours at 37°C and 5% CO2. CFSE: Cells were fixed in 1% formaldehyde and the readings performed in flow cytometer (FACSCalibur, BD). Cell proliferation was determined using flow cytometry and assessed by fluorescence decay of the probe in the gate of CD4+ cells. The frequency of CD25+ Foxp3+ Tregs in the different groups was evaluated in the gate of CD4+ cells. IFN-γ-, IL-10-, and IL-17-producing cells were evaluated in the gate of CD4+ cells as well. Histograms and plots are derived from a representative animal from two independent experiments (n = 5/each assay). (TIF) [file pone.0170205.s001.tif]
